# Supplementary material for: CCN1-Mediated Signaling in Placental Villous Tissues after SARS-CoV-2 Infection in Term Pregnant Women: Implications for Dysregulated Angiogenesis
Source: Curr Issues Mol Biol. 2024 Apr 18;46(4):3533–50. doi: 10.3390/cimb46040221 (PMC11049059; doi:10.3390/cimb46040221)
Supplement: Supplementary file 1 [file cimb-46-00221-s001.zip › cimb-2935490-supplementary.pdf]

Supplementary material: Ma, Duan et al.

Supplementary Table S1. Antibodies used for immunoblotting

Antigen	Species raised in	Company (Catalog No.)	Dilution
**Primary antibody**			
Flt-1	Rabbit	Santa Cruz Biotechnology (sc-316)	1:1000
Endoglin	Goat	R & D systems (AF1097)	1:1000
CCN1	Rabbit	Novus (NB100-356)	1:500
eNOS	Rabbit	Cell Signaling Technology (32027s)	1:1000
TGFβR1	Rabbit	Santa Cruz Technology (sc-398)	1:500
Phospho-NF-κB	Rabbit	Cell Signaling Technology (3033s)	1:1000
NF-κB	Rabbit	Santa Cruz Technology (sc-372)	1:1000
β-Actin	Mouse	Sigma (A3854)	1:200000
**Secondary antibody**			
Rabbit, HRP	Goat	Invitrogen (G21234)	1:5000
Goat, HRP	Donkey	Invitrogen (A15999)	1:5000

Supplementary Table S2. Antibodies used for immunohistochemical/immune-fluorescence staining

Antigen	Species raised in	Company (Catalog No.)	Dilution
**Primary antibody**			
CCN1	Rabbit	Novus (NB100-356)	1:30
Flt-1	Rabbit	Santa Cruz Biotechnology (sc-316)	1:50

Endoglin	Goat	R & D systems (AF1097)	1:50
CD31	Mouse	Dako (M0823)	1:40
CK7	Mouse	Novus (NBP1-22539)	1:80
eNOS	Rabbit	Cell Signaling Technology (32027s)	1:100
TGFβR1	Rabbit	Santa Cruz Technology (sc-398)	1:50
**Secondary antibody**			
Rabbit Alexa-488	Donkey	Abcam (ab150073)	1:200
Mouse Cy3	Goat	Life Technologies (A10521)	1:200
Goat Alexa-488	Donkey	Life Technologies (A11055)	1:200
Rabbit, HRP	Goat	Invitrogen (G21234)	1:100

Supplementary Table S3. SARS-CoV-2 infected vs. non-infected pregnancies: patient cohort for angiogenic protein analysis

Variable	Control (n=14)	SARS-CoV-2 (n=14)	P-value
Maternal age at delivery, years, median (IQR)	31.50 (26.00-35.25)	32.50 (25.75-35.25)	0.7743
Gestational age at sampling, weeks, mean (min and max)	31+5 (28+0-38+5)	38+2 (36+1-41+1)	<0.0001
Gestational age at delivery, weeks, mean (min and max)	37+2 (30+1-40+5)	38+5 (36+5-41+1)	0.1464
Pregnancy BMI before birth, median (IQR)	32.00 (23.75-38.25)	30.00 (27.00-32.00)	0.6817
Cesarean section, no. (%)	7 (50.00)	7 (50.00)	-
Leukocyte, $10^3/mm^3$, mean ±	12.28 ± 6.68	8.22 ± 2.33	0.0421

SD			
CRP, mg/dL, mean ± SD	0.58± 0.96	1.20 ± 1.49	0.4471
Asymptomatic infection, no. (%)	-	8 (57.14)	-
Mild infection, no. (%)	-	6 (42.86)	-
Birth weight, g, mean ± SD	3034.00 ± 664.40	3264 ± 444.70	0.2909
5-min Apgar score, mean (min and max)	9.14 (7-10)	9.24 (8-10)	0.4231

Supplementary Figure S1. Flt-1 and Endoglin were increased in placental villous tissue of SARS-CoV-2 infected women

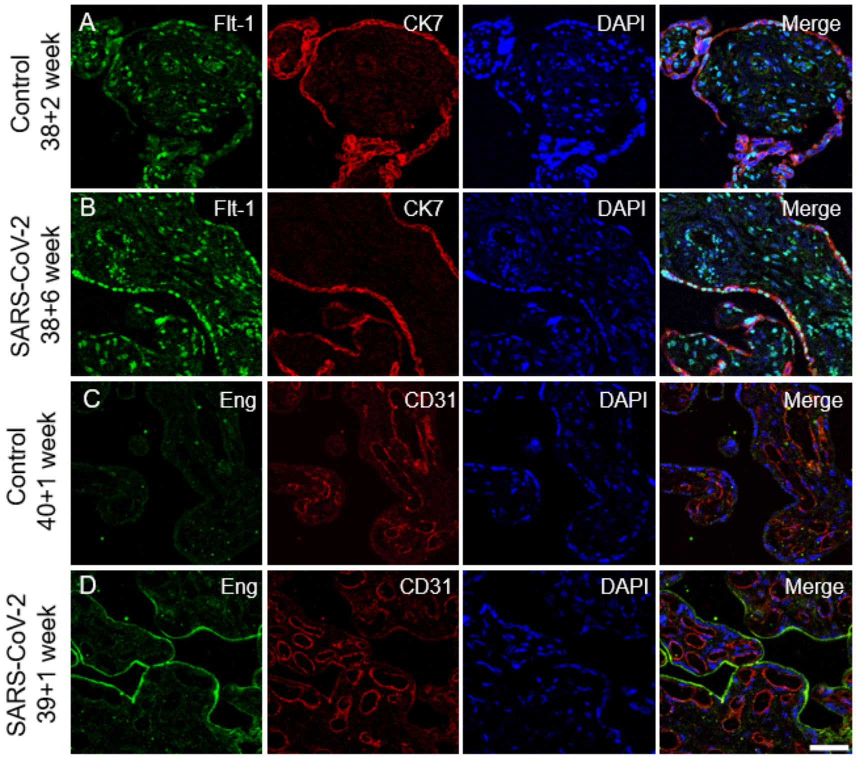

Supplementary Figure S1

Localization of Flt-1 and Endoglin in placental villi of pregnant women with and without SARS-Cov-2. (A-B) Double immunofluorescence staining of Flt-1 and CD31 on placental villus tissue. (C-D) Double immunofluorescence staining of Eng and CK7

on placental villus tissue. Scale bar: 50 μm.

Supplementary materials and methods

1. Placental tissue preparation

Placentas were processed within 4 hours of delivery. The chorionic villous tissue was collected and processed as detailed described [36]. A placental lobule located between the umbilical cord and the edge of the placenta was selected as a middle part, cut with scissors, placed in a culture dish and washed with 1xPBS 3-5 times to remove blood until the washing solution was clear. The decidual basalis and chorionic villous tissue were separated with scissors and tweezers, removing blood vessels and calcifications, then washed again 2-3 times with 1xPBS. Finally, the chorionic villous tissue was frozen at -80°C before used for protein and RNA extraction.

The placental tissue used to perform paraffin sections was processed according to the following procedure. The tissue blocks were fixed with 4% paraformaldehyde then embedded in paraffin. Paraffin-embedded tissues were cut into overnight,5μm sections for histological staining.

2. Cell culture and CCN1 treatment

The trophoblast cell line SGHPL-5 was obtained from the Division of Basic Medical Sciences, St George's University of London (kindly provided by G. Whitley). Cells were routinely cultivated in nutrient mixture F-10 Ham's medium (Sigma, N6908) supplemented with 10% fetal bovine serum (FBS; Gibco, 16000-044), 2mM L-glutamine (Sigma, G8540), 100 U/ml penicillin and 100μg/ml streptomycin (Sigma,

P7539) at 37°C with 5% CO_2. Cells were treated with recombinant human CCN1 (R&D, 4055-CR) at a concentration ranging from 250 to 1000 ng/ml for 24 h.

SGHPL-5 cells were seeded in 6-well plates, changed to serum-free medium before treated with recombinant CCN1. After 24 hours of incubation with CCN1, the supernatant was collected. Suspended cells were removed by centrifuging at 1000 rpm for 10 minutes at 4°C. The medium was subsequently concentrated using centrifugal filter units (Amicon Ultra, UFC500324) according to the manufacturer's instructions. The samples were stored at −80°C until further use.

3. H&E staining

Randomly selected 5 μm placenta sections were stained with a routine hematoxylin and eosin (H&E) stain to verify pathological alterations. Stained sections were scanned by the Aperio CS2 ScanScope slide scanner (Leica, Wetzlar, Germany) at 40×. The acquired files were converted to TIFF format images by Aperio ImageScope (Version 12.4.6.5003, Leica).

4. Quantitative polymerase chain reaction (q-PCR)

The total RNA was extracted from 20mg human placenta tissue using the RNeasy Plus Mini Kit (QIAGEN, 74134) following the manufacturer's protocol. Complementary DNA (cDNA) was synthesized with 1μg RNA as previously described [60]. Relative CCN1 expression levels were quantitated using the qPCR Master Mix SYBR Green (Affymetrix, Santa Clara, USA) and analyzed by an ABI Prism 7300 sequence detector (Applied Biosystems, Foster City, USA) as described before [61]. Relative mRNA expression was determined by standard curve evaluation, normalized to the mean level

of HPRT1 as the housekeeping gene. The following primer sequences were used: CCN1, forward 5'- GTGACGAGGATAGTATCAAGGACC-3', reverse 5'-GTTGGGAAAT GTTCCGGTCTTTA-3'; HPRT1, forward 5'-ACCAGTCAACAGGGGACATAA-3', reverse 5'-AAGCTTGCGACCTTGACC-3'.

5. Collection of Blood Samples and measurement of sFlt-1, PLGF and sEng

Patient blood samples were collected and stored at 4°C within 4 hours to prevent hemolysis. Subsequently, the samples were centrifuged at 2500×g for 10 minutes. Following centrifugation, three to four milliliters of serum were collected then stored at -80°C.

The levels of sFlt-1 and PlGF were quantified using specific Enzyme-Linked immunosorbent assay (ELISA) kits: sFlt-1 (Thermo Fisher Scientific, 845.075) and PlGF-plus (Thermo Fisher Scientific, 859.075), respectively. The measurements were performed on the BRAHMS KRYPTOR compact PLUS machine based on TRACE® Technology (Time-Resolved Amplified Cryptate Emission) (Thermo Fischer Scientific, BRAHMS GmbH, Hennigsdorf, Germany), following the manufacturer's protocol. The measurement of sEng was performed using the Endoglin ELISA kit (R&D Systems, DNDG00), following routine ELISA procedures and the manufacturer's instructions. The detection limit for sFlt-1 was 22 pg/ml, and for PlGF, 3.6 pg/ml. The minimum detectable concentration of sEng was consistently below 0.030 ng/ml. Further details are shown [62].
